# Tumor cell-intrinsic NSUN2 deficiency reprograms macrophages to sensitize non-small cell lung cancer to EGFR inhibitors by reversing immune evasion

**DOI:** 10.1016/j.neo.2026.101326

**Published:** 2026-06-12

**Authors:** Yueqin Wang, Jingyao Wei, Wenbin Xu, Yu Zhang, Luyao Feng, Lizhen Zhang, Shuaibing Liu, Xin Tian

**Affiliations:** a Department of Pharmacy, the First Affiliated Hospital of Zhengzhou University, Zhengzhou 450052, China; b Henan Key Laboratory of Precision Clinical Pharmacy, Zhengzhou University, Zhengzhou 450052, China

**Keywords:** Non-small cell lung cancer, EGFR-TKI resistance, RNA m^5^C modification, Tumor immune evasion, Macrophage polarization

## Abstract

Despite EGFR-tyrosine kinase inhibitors (EGFR-TKI) have improved outcomes in non-small cell lung cancer (NSCLC), acquired resistance remains a challenge. While RNA methylation has been implicated in drug resistance, its role in EGFR-TKI resistance through immune regulation remains unexplored. Here, we established gefitinib- and osimertinib-resistant NSCLC cells and found that NSUN2 knockdown did not affect proliferation or drug sensitivity under immunodeficient conditions. Conversely, under immunocompetent conditions, NSUN2 knockdown suppressed proliferation and restored EGFR-TKI sensitivity by converting ‘cold’ resistant tumors to ‘hot’ tumors. Mechanistically, NSUN2 knockdown promoted macrophage migration and chemokine expression, and enhanced M0-to-M1 polarization, with opposite effects observed upon NSUN2 overexpression. Integrative RNA Bis-seq and RNA-seq identified the E3 ubiquitin ligase TRIM29 as a key target downregulated upon NSUN2 knockdown and upregulated upon overexpression. Consistently, TRIM29 depletion phenocopied NSUN2 knockdown by promoting M1 polarization and macrophage migration. These findings identify NSUN2 as a driver of immune evasion and acquired EGFR-TKI resistance through epigenetic regulation of macrophage infiltration and polarization.

## Introduction

Non-small cell lung cancer (NSCLC) is the leading cause of cancer-related mortality worldwide [1,2]. Although EGFR-tyrosine kinase inhibitor (EGFR-TKI) targeted therapy has significantly prolonged survival in patients with driver mutations [[3], [4], [5]], acquired resistance remains a critical clinical challenge [6]. Notably, EGFR-mutant NSCLC typically exhibits characteristics of low immunogenicity and a non-inflammatory tumor microenvironment, contributing to its limited response to immunotherapy [7,8]. However, accumulating evidence indicates that EGFR-TKI can reshape the tumor immune microenvironment (TIME) by upregulating PD-L1 expression, increasing tumor mutation burden, and enhancing immune cell activity [9,10]. These mechanistic insights have established immunotherapy as a viable therapeutic strategy for NSCLC patients after EGFR-TKI failure [11,12], highlighting the critical importance of elucidating how tumor immune evasion mediates EGFR-TKI resistance to optimize treatment strategies for this patient population.

RNA methylation modifications play important roles in regulating the TIME. While m^6^A modification participates in immune evasion by regulating immune cell function, cytokine secretion, and metabolic reprogramming [13], the immune regulatory functions of m^5^C modification remain largely unexplored. NSUN2 (NOP2/Sun RNA Methyltransferase 2), the key enzyme catalyzing RNA m^5^C deposition, has been established as a driver of tumor progression and drug resistance through its regulation of RNA stability and translation [14,15]. Notably, recent evidence indicates that NSUN2 may also facilitate immune evasion via m^5^C-dependent stabilization of PD-L1 mRNA in liver cancer [16,17]. Whether NSUN2 modulates tumor immunity to influence EGFR-TKI resistance, however, remains to be elucidated.

Building upon our previous finding that NSUN2-mediated m^5^C modification drives intrinsic EGFR-TKI resistance through the NSUN2-YBX1-QSOX1 axis [18], this study demonstrates that NSUN2 also governs acquired resistance to EGFR-TKI through immune-dependent mechanisms. Specifically, NSUN2 knockdown restored EGFR-TKI sensitivity exclusively under immune-competent conditions by converting immunologically ‘cold’ resistant tumors to ‘hot’ tumors. Mechanistically, NSUN2 negatively regulates both macrophage infiltration and M1 polarization, establishing it as a critical modulator of the tumor immune microenvironment in EGFR-TKI-resistant NSCLC.

## Results

### NSUN2 fails to regulate EGFR-TKI sensitivity in NSCLC cells under immunodeficient conditions

**Fig. 1:**
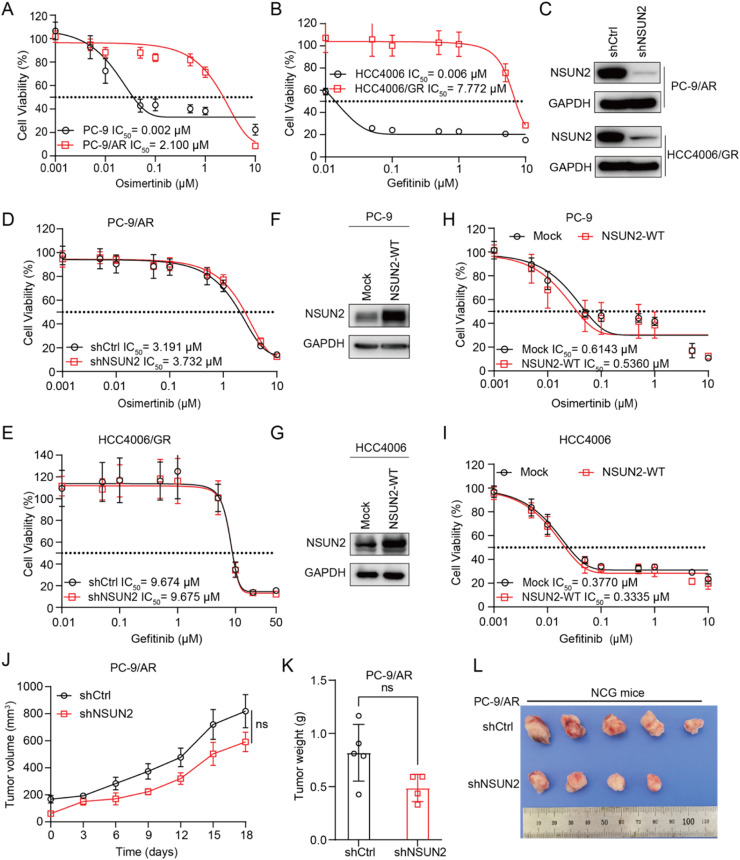
Knockdown of NSUN2 does not affect the proliferation or EGFR-TKI sensitivity of acquired resistant cells under immunodeficient conditions. (A, B) CCK-8 assay assessing osimertinib sensitivity in PC-9 and PC-9/AR cells (A) and gefitinib sensitivity in HCC4006 and HCC4006/GR cells (B). (C) Western blot detection of NSUN2 knockdown efficiency in PC-9/AR and HCC4006/GR cells. (D, E) CCK-8 assay assessing osimertinib sensitivity in PC-9/AR cells (D) and gefitinib sensitivity in HCC4006/GR cells (E) with NSUN2 knockdown. (F, G) Western blot was used to detect stable NSUN2 overexpression efficiency at protein levels in PC-9 (F) and HCC4006 (G) cells, respectively. (H) CCK-8 assay was performed to assess the effect of NSUN2 overexpression on osimertinib sensitivity in PC-9 NSUN2-WT cells. (I) CCK-8 assay was performed to assess the effect of NSUN2 overexpression on gefitinib sensitivity in HCC4006 NSUN2-WT cells. (J-L) Tumor growth curve (J), tumor weight (K), and representative images (L) of subcutaneous xenografts from PC-9/AR shCtrl (n = 5) and PC-9/AR shNSUN2 (n = 4) cells in NCG immunodeficient mice. Data are presented as mean ± SD. ns indicates no significant difference. Mock, empty vector control. NSUN2-WT, wild type NSUN2.

To investigate the role of NSUN2 in NSCLC acquired resistance, we established gefitinib-resistant (HCC4006/GR) and osimertinib-resistant (PC-9/AR) cell lines using a stepwise dose-escalation method (Fig. 1A, B). NSUN2 was stably knocked down in both resistant cell lines via lentivirus-mediated shRNA, with knockdown efficiency confirmed by Western blot (Fig. 1C). Under immunodeficient conditions *in vitro*, NSUN2 knockdown had no significant effect on EGFR-TKI sensitivity, as evidenced by unchanged IC_50_ values for osimertinib in PC-9/AR cells (Fig. 1D) and gefitinib in HCC4006/GR cells (Fig. 1E). To further validate these findings, we overexpressed NSUN2 in sensitive cell lines PC-9 and HCC4006. Compared with the empty vector control (Mock), protein levels of NSUN2 were significantly upregulated in the NSUN2-WT group (Fig. 1F, G). Subsequent CCK-8 assays showed that NSUN2 overexpression in PC-9 and HCC4006 cells did not significantly alter their IC_50_ values for EGFR-TKI (osimertinib and gefitinib), which remained comparable to those of parental cells (Fig. 1H, I).

To extend these observations *in vivo*, we performed experiments using NCG severely immunodeficient mice. NSUN2 knockdown did not significantly inhibit subcutaneous tumor growth, with no differences in tumor volume or weight compared to controls (Fig. 1J-L). Collectively, these findings demonstrate that neither knockdown nor overexpression of NSUN2 affects EGFR-TKI sensitivity or tumor growth in NSCLC cells under immunodeficient conditions.

### NSUN2 is associated with tumor immune response in EGFR-TKI-resistant NSCLC based on transcriptome profiling

**Fig. 2:**
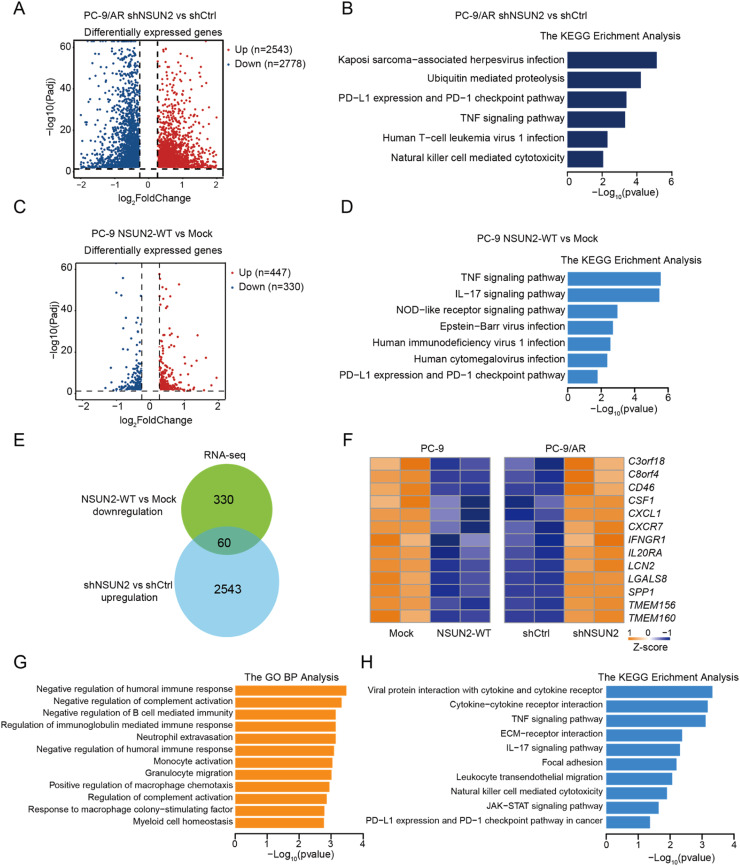
Transcriptome analysis reveals an association between NSUN2 expression and immune response in EGFR-TKI-resistant NSCLC. (A, B) Effects of NSUN2 knockdown in PC-9/AR resistant cells: (A) Volcano plot of differentially expressed genes; (B) KEGG pathway enrichment analysis of upregulated genes. (C, D) Effects of NSUN2 overexpression in PC-9 sensitive cells: (C) Volcano plot of differentially expressed genes; (D) KEGG pathway enrichment analysis of downregulated genes. (E) Venn diagram showing the overlap between significantly upregulated genes upon NSUN2 knockdown and significantly downregulated genes upon NSUN2 overexpression. (F) Heatmap clustering analysis of selected differentially expressed genes from (F). (G) GO enrichment analysis of differentially expressed genes. (H) KEGG enrichment analysis of differentially expressed genes.

To elucidate the mechanism by which NSUN2 regulates EGFR-TKI acquired resistance, we performed transcriptome sequencing following NSUN2 knockdown in resistant cells and overexpression in sensitive cells. RNA-seq analysis revealed that NSUN2 knockdown in PC-9/AR cells upregulated 2,543 genes, with significant enrichment in tumor immunity-related pathways including PD-L1 signaling, TNF-α signaling, and NK cell-mediated cytotoxicity (Fig. 2A, B). Conversely, NSUN2 overexpression in PC-9 cells downregulated 330 genes including the same immune-related gene set, with suppression of TNF signaling, IL-17 signaling, and PD-1/PD-L1 immune checkpoint pathways (Fig. 2C, D). Intersection analysis identified 60 common genes between these reciprocal datasets (Fig. 2E), predominantly immune-related genes (C3orf18, CD46, CSF1, CXCL1, CXCR7, IFNGR1, LCN2, etc.) (Fig. 2F). GO and KEGG enrichment analyses revealed these genes were involved in complement activation, B cell-mediated immunity, monocyte activation, and leukocyte migration (Fig. 2G, H). These transcriptomic findings indicate that NSUN2 suppresses tumor immune responses, prompting us to investigate whether this immune modulation functionally impacts EGFR-TKI resistance.

### NSUN2 knockdown suppresses cell proliferation of resistant cells in the presence of PBMCs

**Fig. 3:**
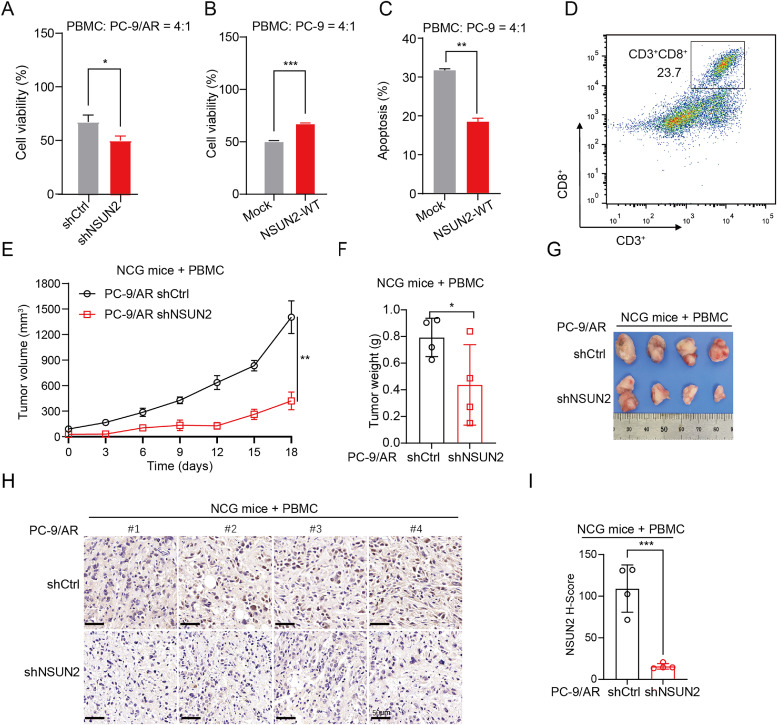
NSUN2 knockdown suppresses the proliferation of resistant cells upon immune reconstitution. (A, B) PBMCs were co-cultured with tumor cells at a 4:1 ratio, and CCK-8 assay was performed to assess the effects of NSUN2 knockdown on resistant cell proliferation (A) and NSUN2 overexpression on sensitive cell proliferation (B). (C) Flow cytometry analysis of apoptosis in cells overexpressing NSUN2 under PBMC co-culture conditions. (D) Flow cytometry analysis of CD3⁺CD8⁺ T cell proportion in mouse spleen. (E) Growth curve of subcutaneous xenografts (n = 4). (F)Tumor weight comparison. (G) Representative images of xenograft tumors. (I, H) Representative images of NSUN2 staining (scale bar: 50 μm) (H) and quantitative analysis of staining intensity (I) in tumor tissues. Data are presented as mean ± SD. *P < 0.05, **P < 0.01, ***P < 0.001.

To functionally validate the immunomodulatory effects of NSUN2 on EGFR-TKI resistance, we established an *in vitro* co-culture system with peripheral blood mononuclear cells (PBMCs). Under these immunocompetent conditions, NSUN2 knockdown significantly suppressed proliferation of PC-9/AR resistant cells (Fig. 3A), whereas NSUN2 overexpression promoted proliferation of PC-9 sensitive cells (Fig. 3B) and inhibited their apoptosis (Fig. 3C). To validate these findings *in vivo*, we established a humanized immune-reconstituted NCG mouse model by injecting human PBMCs (1 × 10⁷ cells per mouse) via tail vein, followed by subcutaneous inoculation of PC-9/AR cells 3 days later. Successful immune reconstitution was confirmed by increased CD3⁺CD8⁺ T cell proportions in spleen (Fig. 3D). In this immunocompetent setting, NSUN2 knockdown significantly suppressed *in vivo* tumor growth, as evidenced by reduced tumor volume and weight (Fig. 3E-G), with confirmed reduction of NSUN2 protein in tumor tissues (Fig. 3H-I). Collectively, these findings demonstrate that NSUN2 knockdown inhibits proliferation of EGFR-TKI-resistant cells specifically under immunocompetent conditions.

### NSUN2 knockdown enhances EGFR-TKI sensitivity in resistant cells upon immune reconstitution

**Fig. 4:**
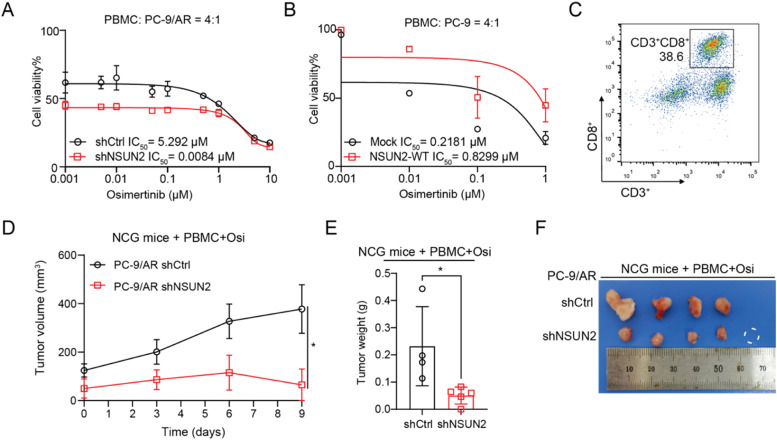
NSUN2 knockdown enhances EGFR-TKI sensitivity in resistant cells upon immune reconstitution. (A, B) CCK-8 assay was performed to evaluate changes in osimertinib sensitivity following NSUN2 knockdown in PC-9/AR cells (A) or NSUN2 overexpression in PC-9 cells (B) under PBMC co-culture conditions. (C) Flow cytometry analysis of CD3⁺CD8⁺ T cell proportion in mouse spleen. (D) Growth curve of subcutaneous xenografts of PC-9/AR shCtrl (n = 4) and PC-9/AR shNSUN2 (n = 5) cells. (E) Comparison of tumor weight. (F) Representative images of subcutaneous xenografts. Data are presented as mean ± SD. *P < 0.05. Osi, osimertinib.

We further examined whether NSUN2 modulates EGFR-TKI sensitivity through tumor-immune cell interactions. In PBMC co-culture experiments, NSUN2 knockdown significantly reduced the IC_50_ of osimertinib in PC-9/AR cells, whereas NSUN2 overexpression increased the IC_50_ in PC-9 cells (Fig. 4A, B), demonstrating that NSUN2 regulates EGFR-TKI sensitivity in an immune-dependent manner. To validate this *in vivo*, we established a subcutaneous xenograft model using PC-9/AR cells in humanized immune-reconstituted NCG mice, followed by osimertinib treatment (5 mg/kg/d) for 2 weeks. Successful immune reconstitution was confirmed by elevated CD3⁺CD8⁺ T cell proportions in spleen (Fig. 4C). Notably, the combination of NSUN2 knockdown and osimertinib markedly suppressed tumor growth compared with osimertinib alone, as evidenced by significantly reduced tumor volume and weight (Fig. 4D-F). Collectively, these findings establish that NSUN2 knockdown enhances EGFR-TKI sensitivity specifically under immunocompetent conditions.

### NSUN2 suppresses macrophage infiltration in EGFR-TKI-resistant NSCLC

**Fig. 5:**
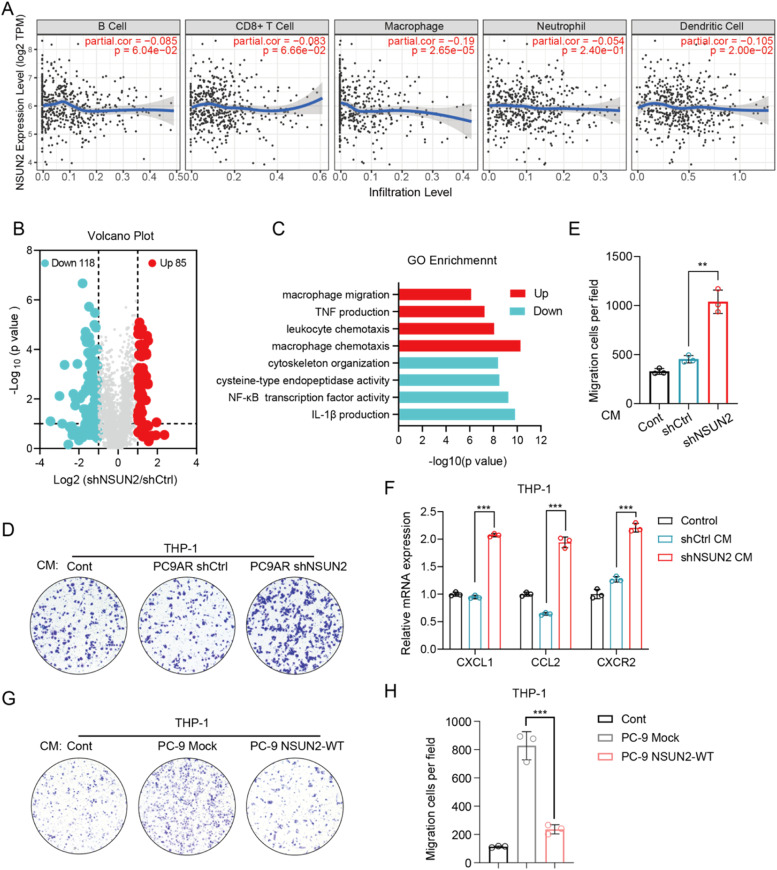
NSUN2 suppresses macrophage infiltration in EGFR-TKI-resistant NSCLC. (A) Correlation analysis between NSUN2 expression levels and infiltration of major immune cell types in lung cancer patients from the TCGA-LUAD database. (B, C) Proteomic analysis of PC-9/AR xenografts upon NSUN2 knockdown. Volcano plot of differentially expressed proteins in NSUN2 knockdown group versus control group (B); GO pathway enrichment analysis of differentially expressed proteins (C). (D, E) Transwell assay assessing the effect of conditioned medium from PC-9/AR cells transfected with control shRNA (shCtrl) or NSUN2-targeting shRNA (shNSUN2) on THP-1 macrophage migration (D) and its quantitative analysis (E). (F) qRT-PCR analysis of chemotaxis-related gene expression in THP-1 macrophages treated with conditioned medium from NSUN2-knockdown PC-9/AR cells. (G, H) Transwell assay assessing the effect of conditioned medium from PC-9 cells transfected with empty vector (Mock) or wild-type NSUN2 (NSUN2-WT) on THP-1 macrophage migration (G), with corresponding quantitative analysis (H). Data are presented as mean ± SD. **P < 0.01, ***P < 0.001. CM, conditioned medium; Cont, control medium.

Based on the above findings, we focused on elucidating the mechanism by which NSUN2 influences the lung cancer immune microenvironment. Analysis of the TCGA-LUAD database revealed that NSUN2 expression levels were significantly negatively correlated with macrophage infiltration in lung cancer tissues (Fig. 5A). Consistently, proteomic analysis of xenograft tissues showed that proteins upregulated upon NSUN2 knockdown were significantly enriched in signaling pathways associated with macrophage migration and chemotaxis (Fig. 5B, C). These bioinformatic predictions were functionally validated by *in vitro* transwell assays, which demonstrated that conditioned medium from NSUN2-knockdown resistant cells significantly promoted macrophage migration (Fig. 5D, E), accompanied by upregulated mRNA expression of chemokines CXCL1, CCL2 and their receptor CXCR2 (Fig. 5F). Conversely, conditioned medium from NSUN2-overexpressing cells exerted the opposite effect (Fig. 5G, H). Collectively, these findings establish that NSUN2 in resistant cells negatively regulates macrophage infiltration, thereby influencing EGFR-TKI resistance.

### NSUN2 negatively regulates M1 macrophage polarization

**Fig. 6:**
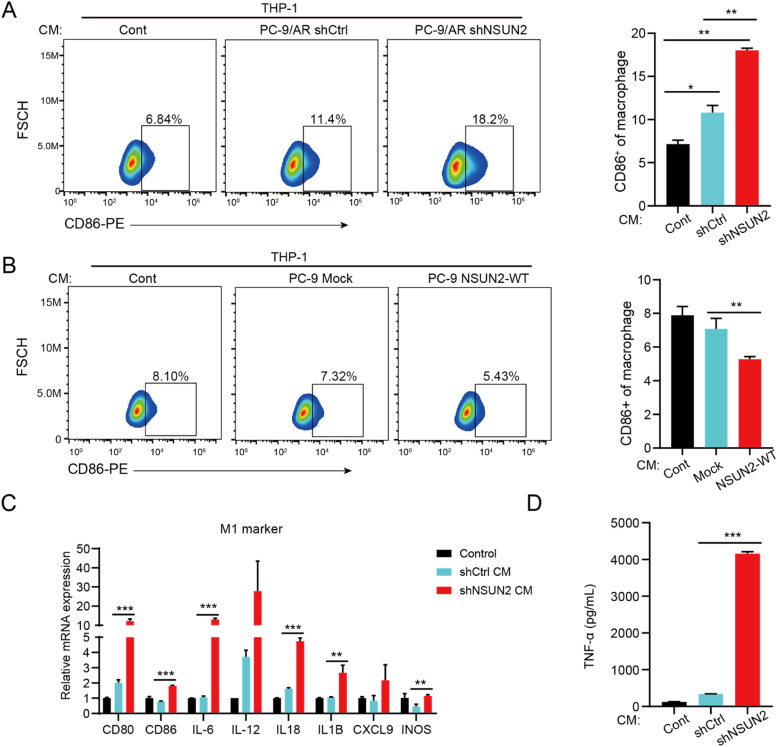
NSUN2 negatively regulates M1 macrophage polarization. (A) Flow cytometry analysis of macrophage phenotypes following treatment with conditioned medium from NSUN2-knockdown cells. Left: Representative flow cytometry plots; Right: Quantitative statistical analysis. (B) Flow cytometry analysis of macrophage phenotypes following treatment with conditioned medium from NSUN2-overexpressing cells. (C) qRT-PCR analysis of M1 surface marker mRNA levels in macrophages treated with conditioned medium from NSUN2-knockdown PC-9/AR cells. (D) ELISA detection of TNF-α secretion levels in macrophages activated by conditioned medium from NSUN2-knockdown cells. Data are presented as mean ± SD. *P < 0.05, **P < 0.01, ***P < 0.001.

The above results demonstrated that NSUN2 knockdown significantly promotes macrophage infiltration into the tumor microenvironment. Building upon this finding, we further investigated the effect of NSUN2 on the phenotypic polarization of macrophages. Flow cytometry analysis showed that conditioned medium from NSUN2-knockdown cells significantly promoted M1 polarization (increased CD86⁺ proportion) (Fig. 6A), whereas conditioned medium from NSUN2-overexpressing cells produced the opposite effect (Fig. 6B). qRT-PCR analysis further showed upregulation of M1 markers (iNOS, TNF-α) (Fig. 6C), and ELISA demonstrated enhanced TNF-α secretion by macrophage cells treated with NSUN2-knockdown conditioned medium (Fig. 6D). Collectively, these findings suggest that NSUN2 deficiency in resistant cells promotes macrophage polarization toward the M1 phenotype.

### TRIM29 was identified as a potential target of NSUN2-mediated EGFR-TKI resistance

**Fig. 7:**
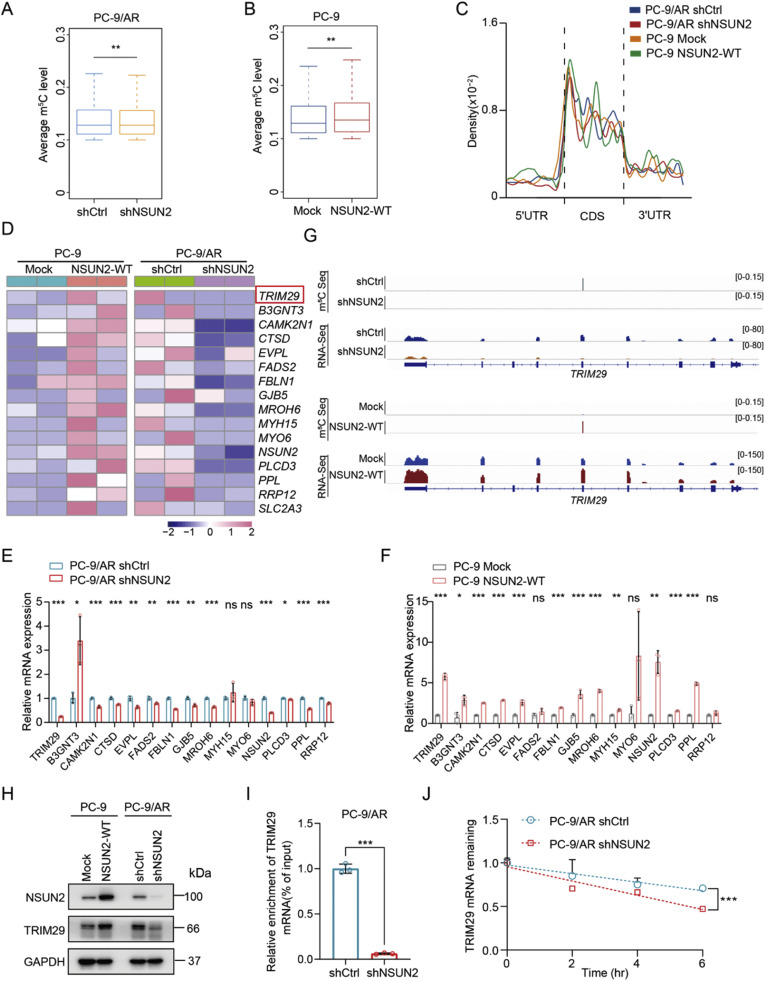
Epi-transcriptome analysis identifies TRIM29 as an m^5^C-modified target in resistant NSCLC cells. (A, B) Box plots depicting the average m^5^C level in PC-9/AR versus PC-9/AR shNSUN2 cells (A) and PC-9 versus PC-9 NSUN2-WT cells (B). (C) Metagene analysis showing the distribution of m^5^C peaks across mRNA transcript regions. (D) Clustering heatmap displaying candidate target genes identified by integrative analysis of m^5^C sequencing and transcriptome sequencing. (E) qRT-PCR analysis of candidate target gene expression in PC-9/AR cells with NSUN2 knockdown. (F) qRT-PCR analysis of candidate target gene expression in PC-9 cells with stable NSUN2 overexpression. (G) IGV analysis showed that changes in mRNA expression and m^5^C levels of TRIM29 in PC-9/AR and PC-9 cells upon NSUN2 knockdown or overexpression. (H) Western blot analysis of TRIM29 protein levels in PC-9 NSUN2-WT and PC-9/AR shNSUN2 cells. (I) Purified mRNA was immunoprecipitated by anti-m^5^C antibody and the m^5^C levels of TRIM29 in PC-9/AR were analyzed by qRT-PCR. (J) PC-9/AR cells transfected with shNSUN2 were treated with actinomycin-D (5 μg/mL) for the indicated time points and TRIM29 mRNA half-life was analyzed by qRT-PCR. Data are presented as mean ± SD. *P < 0.05, **P < 0.01, ***P < 0.001.

To identify NSUN2 targets underlying acquired EGFR-TKI resistance in NSCLC, transcriptome-wide m^5^C profiling was conducted using RNA-BisSeq. Quantification revealed that NSUN2 depletion in PC-9/AR cells significantly decreased global m^5^C levels, whereas ectopic NSUN2 expression in PC-9 cells markedly increased m^5^C abundance (Fig. 7A, B). m^5^C peaks were predominantly localized within coding sequences (CDS) (Fig. 7C and Fig. S1A, B). Hierarchical clustering of differentially methylated sites revealed distinct epitranscriptomic signatures between NSUN2-manipulated and control cells (Fig. S1C, D). Functional annotation showed that hypomethylated genes upon NSUN2 knockdown were enriched in oncogenic pathways including Wnt signaling and ubiquitin-mediated proteolysis (Fig. S1E), while hypermethylated genes following NSUN2 overexpression were associated with protein biosynthesis, intracellular transport, and ubiquitin-dependent catabolic processes (Fig. S1F).

To identify specific target genes regulated by NSUN2-mediated m^5^C modification, we analyzed the intersection of transcriptomic and epitranscriptomic data. We identified 836 genes (1,642 sites) exhibiting both downregulated mRNA expression and m^5^C hypomethylation in PC-9/AR cells upon NSUN2 knockdown (Fig. S1G), and 183 genes (365 sites) showing upregulated mRNA expression and m^5^C hypermethylation in PC-9 cells upon NSUN2 overexpression (Fig. S1H). The intersection of these two gene sets yielded 16 candidate target genes (Fig. 7D). Among these candidates, qRT-PCR analysis revealed that TRIM29 exhibited the most pronounced changes, with its mRNA expression significantly downregulated in PC-9/AR cells upon NSUN2 knockdown and markedly upregulated in PC-9 cells upon NSUN2 overexpression (Fig. 7E, F). Integrative Genomics Viewer (IGV) analysis confirmed that both mRNA expression and m^5^C levels of TRIM29 decreased substantially in PC-9/AR cells with NSUN2 knockdown, whereas they increased obviously in PC-9 cells with NSUN2 overexpression (Fig. 7G). Western blot analysis further demonstrated that NSUN2 knockdown markedly suppressed TRIM29 protein expression, whereas NSUN2 overexpression significantly elevated its protein level (Fig. 7H).

To elucidate the mechanism underlying NSUN2-mediated regulation of TRIM29, methylated RNA immunoprecipitation (MeRIP) combined with qRT-PCR was performed. The results indicated that NSUN2 knockdown significantly decreased the m^5^C enrichment level of TRIM29 mRNA in PC-9/AR cells compared with control (Fig. 7I). Furthermore, RNA stability assays revealed that NSUN2 knockdown substantially reduced the half-life and stability of TRIM29 mRNA (Fig. 7J). Collectively, these findings suggest that TRIM29 is a potential target of NSUN2-mediated m^5^C modification, and that NSUN2 may enhance TRIM29 expression through m^5^C-dependent stabilization of its transcript, thereby contributing to acquired resistance to EGFR-TKI in NSCLC.

### TRIM29 knockdown promotes M1 macrophage polarization and macrophage migration in EGFR-TKI-resistant cells

**Fig. 8:**
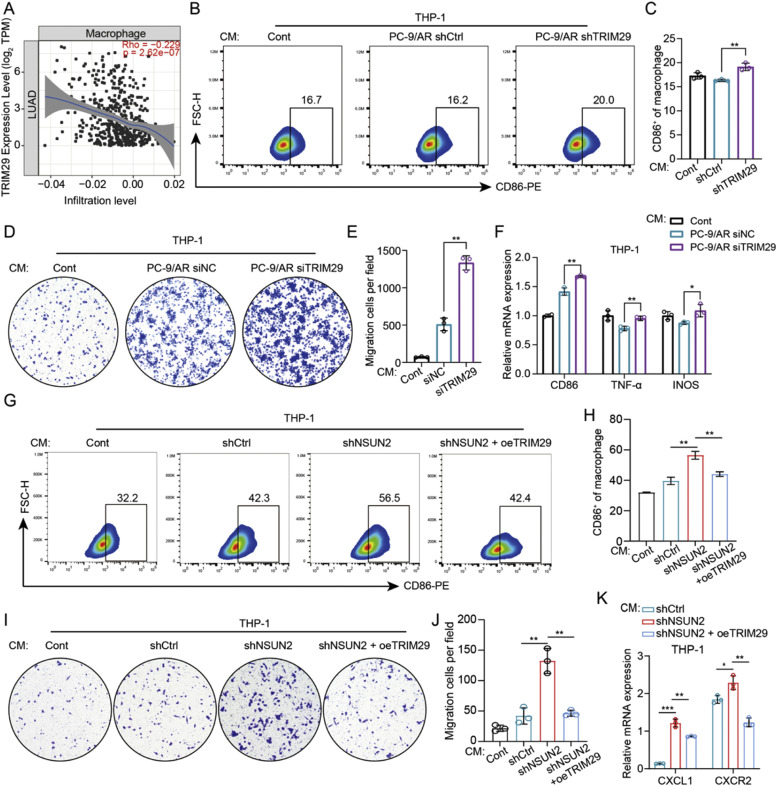
Knockdown of TRIM29 in resistant NSCLC cells promotes M1 macrophage polarization and migration. (A) Correlation analysis between TRIM29 expression levels and infiltration of macrophages in lung cancer patients from the TCGA-LUAD database. (B, C) Flow cytometry analysis of M1 polarization in THP-1 macrophages treated with conditioned medium from PC-9/AR cells with TRIM29 knockdown: representative flow cytometry plots (B) and quantitative statistics (C). (D, E) Transwell assay of THP-1 macrophage migration in response to conditioned medium from PC-9/AR cells with TRIM29 knockdown (D) and quantitative analysis (E). (F) qRT-PCR analysis of M1 marker mRNA expression in THP-1 macrophages treated with conditioned medium from PC-9/AR cells with TRIM29 knockdown. (G, H) Flow cytometry analysis of M1 polarization in THP-1 macrophages following treatment with conditioned medium from PC-9/AR cells pre-treated with shNSUN2 and stably transfected with TRIM29 (oe TRIM29). Representative plots are shown in (G), and quantitative data are shown in (H). (I, J) Transwell assay of THP-1 macrophage migration in response to conditioned medium from PC-9/AR cells pre-treated with shNSUN2 and stably transfected with TRIM29 (oe TRIM29) (I) and quantitative analysis (J). (K) qRT-PCR analysis of chemotaxis-related gene expression in THP-1 macrophages treated with conditioned medium from PC-9/AR cells pre-treated with shNSUN2 and stably transfected with TRIM29 (oe TRIM29). Data are presented as mean ± SD. Statistical significance was determined by two-tailed unpaired Student's t-test. *P < 0.05, **P < 0.01.

Given that NSUN2 promotes EGFR-TKI resistance via TRIM29, we next investigated whether TRIM29 itself could regulate macrophage infiltration and polarization. Analysis of the TCGA-LUAD database revealed that TRIM29 expression levels were significantly and negatively correlated with macrophage infiltration in lung cancer tissues (Fig. 8A). Consistent with the effect of NSUN2 knockdown, flow cytometry analysis showed that conditioned medium from TRIM29-depleted cells significantly promoted M0 macrophage polarization toward the M1 phenotype, as evidenced by an increased proportion of CD86^+^ macrophages (Fig. 8B, C). Transwell assays further demonstrated that conditioned medium from TRIM29-knockdown cells enhanced macrophage migration capacity (Fig. 8D, E). Additionally, qRT-PCR analysis confirmed that conditioned medium from TRIM29-depleted cells markedly upregulated mRNA expression of M1-associated markers, including CD86, TNF-α and iNOS, in macrophages (Fig. 8F). Notably, TRIM29 overexpression (oe-TRIM29) largely reversed the enhanced M1 polarization and increased macrophage migration induced by NSUN2 knockdown (Fig. 8G-J), concomitant with downregulated mRNA expression of the chemokine CXCL1 and its receptor CXCR2 (Fig. 8K). Collectively, these findings indicate that NSUN2 regulates macrophage infiltration and polarization through TRIM29, thereby promoting acquired resistance to EGFR-TKI.

### NSUN2 and TRIM29 are associated with NSCLC progression and macrophage infiltration

**Fig. 9:**
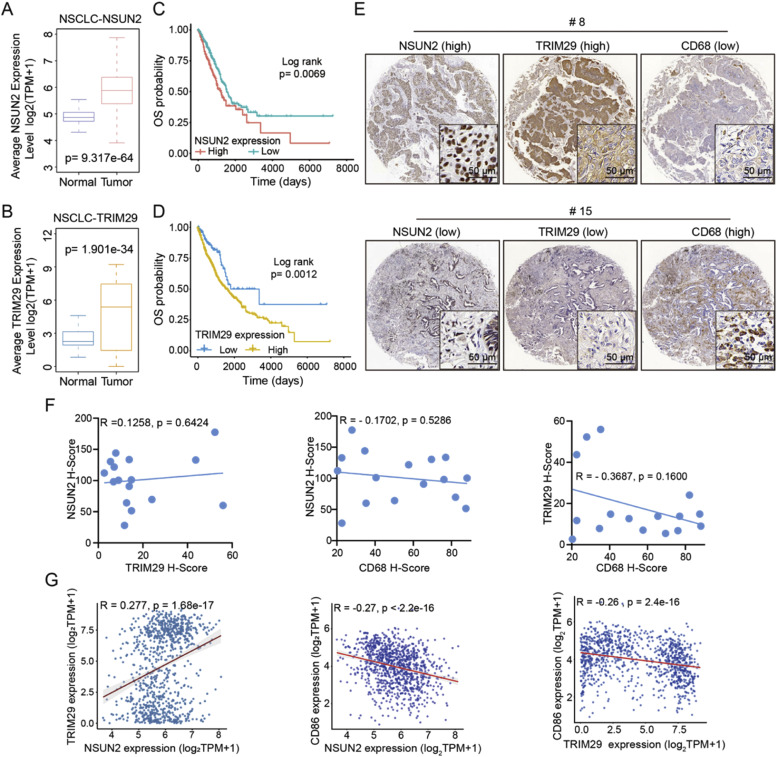
NSUN2 and TRIM29 are associated with NSCLC progression and macrophage infiltration. (A, B) Analysis of TCGA data showing mRNA expression levels of NSUN2 (A) and TRIM29 (B) in normal lung tissues and NSCLC tissues. (C, D) Kaplan–Meier survival curves from the TCGA-NSCLC dataset demonstrating that high expression of NSUN2 (C) and TRIM29 (D) is associated with poorer overall survival. P values were calculated using log-rank tests. (E) Immunohistochemical staining of NSUN2, TRIM29, and CD68 in NSCLC tissue microarrays from 16 paired cases; representative images are shown. Scale bars, 50 μm. (F) Correlation analysis among NSUN2, TRIM29, and CD68 expression in the NSCLC cohort. (G) Correlation analyses among NSUN2, TRIM29, and CD86 expression in the TCGA cohort.

To investigate the role of NSUN2/TRIM29 in NSCLC progression, we first analyzed TCGA data and found that both NSUN2 and TRIM29 were significantly upregulated in NSCLC tissues compared to adjacent normal tissues (Fig. 9A, B). Kaplan-Meier survival analysis further demonstrated that elevated expression of either NSUN2 or TRIM29 was associated with poorer overall survival (OS) (Fig. 9C, D). Building on our mechanistic findings that NSUN2 modulates macrophage infiltration via TRIM29 in NSCLC, we next examined whether this regulatory axis is reflected in clinical samples. Immunohistochemistry (IHC) analysis of NSCLC tissues indicated that NSUN2 and TRIM29 expression levels were suggestive of a positive correlation, whereas both proteins exhibited a suggestive negative correlation with the proportion of CD68-positive macrophages (Fig. 9E, F). These observations were further corroborated by TCGA analysis, which revealed a positive correlation between NSUN2 and TRIM29, alongside a negative correlation between NSUN2/TRIM29 expression and CD86 levels (Fig. 9G). Collectively, these clinical findings support our *in vitro* observations, demonstrating that NSUN2 and TRIM29 are coordinately upregulated in NSCLC tissues, where their elevated expression not only predicts poor survival but also shows a potential association with reduced macrophage infiltration.

## Discussion

Although EGFR-TKI have significantly improved clinical outcomes in NSCLC, acquired resistance remains a major obstacle. While RNA m^5^C modification has been implicated in tumor drug resistance, current studies have predominantly focused on its cell-autonomous regulatory effects, with limited investigation into its capacity to reshape the immune microenvironment and drive therapeutic resistance. Here, we demonstrate that the regulatory effect of NSUN2 on EGFR-TKI resistance is critically immune-dependent. Specifically, NSUN2 deficiency effectively suppressed xenograft growth and restored EGFR-TKI sensitivity only under immunocompetent conditions, with no such effects observed under immunodeficient conditions. Mechanistically, NSUN2 deficiency in lung cancer cells significantly promoted macrophage infiltration into the tumor microenvironment and induced their polarization toward the anti-tumor M1 phenotype, thereby converting resistant cells from ‘cold’ to ‘hot’ tumors, whereas NSUN2 overexpression in sensitive cells drove the opposite transition. These findings establish that elevated NSUN2 expression in resistant NSCLC cells fosters an immunosuppressive tumor microenvironment, promoting immune evasion and ultimately conferring EGFR-TKI resistance.

The TIME plays an increasingly recognized central role in mediating EGFR-TKI resistance, with the complex interplay between TIME and tumor cells emerging as a forefront area of investigation in resistance mechanisms. Studies have shown that during EGFR-TKI treatment, tumor cells secrete paracrine factors such as IL-6 and TGF-β, which not only recruit substantial numbers of regulatory T cells (Tregs) and myeloid-derived suppressor cells (MDSCs), but also drive tumor-associated macrophages (TAMs) toward M2 polarization, ultimately reshaping the TIME into an immunosuppressive state [[19], [20], [21]]. Conversely, this immunosuppressive TIME can feedback to drive the development of EGFR-TKI resistance [[22], [23], [24], [25]]. In this study, we demonstrated that NSUN2 knockdown in resistant cells significantly upregulated immune-related genes and activated tumor immunity-related signaling pathways, whereas NSUN2 overexpression in sensitive cells produced the opposite effects. Intersection analysis of differentially expressed genes further revealed NSUN2’s broad involvement in immune cell activation, proliferation and migration, regulation of tumor microenvironment immune responses, and inflammatory cytokine release. Functionally, these molecular alterations translated into profound phenotypic changes, with reduced NSUN2 expression converting resistant cells from immunologically ‘cold’ to ‘hot’ tumors and NSUN2 overexpression driving the reverse transition in sensitive cells. Collectively, these findings establish NSUN2 as a critical epigenetic regulator of tumor immune evasion and TIME remodeling in NSCLC, highlighting its potential as a therapeutic target to overcome EGFR-TKI resistance through immune modulation.

Immune cell infiltration is a critical determinant of clinical therapeutic efficacy. Previous studies have demonstrated that RNA m^5^C modification participates in regulating immune cell infiltration within the TIME. For instance, elevated expression of the m^5^C reader protein ALYREF correlates with reduced CD8⁺ T cell infiltration [26], while high YBX1 expression is associated with M2 macrophage infiltration and T cell exhaustion [27]. In the present study, we found that NSUN2 expression levels in lung cancer tissues were significantly negatively correlated with macrophage infiltration, and NSUN2 knockdown effectively enhanced macrophage recruitment into tumor tissues. Macrophages exhibit substantial phenotypic plasticity within the TIME and are generally classified into pro-inflammatory, anti-tumor M1 macrophages and anti-inflammatory, pro-tumorigenic M2 macrophages based on their functional characteristics. Recent studies have highlighted the important role of epigenetic modifications in regulating macrophage polarization. For example, histone modifications such as H3K27me3 demethylation promote M1 polarization [28], whereas upregulation of DNA methyltransferase DNMT3b is associated with M2 polarization [29]. Additionally, the RNA m^6^A methyltransferase METTL3 facilitates M1 polarization by stabilizing Stat1 mRNA [30]. Here, we demonstrated that NSUN2 knockdown in resistant cells not only induced unpolarized M0 macrophages to polarize toward the M1 phenotype but also promoted the re-polarization of established M2 macrophages back to the M1 state. These findings indicate that NSUN2, as an epitranscriptomic regulator, can remodel the TIME by modulating macrophage infiltration and reprogramming macrophage states, thereby driving the conversion of immunologically ‘cold’ tumors to ‘hot’ tumors. Therefore, elucidating whether NSUN2 regulates tumor cell-secreted proteins through m⁵C modification to mediate tumor-macrophage communication and thereby ‘educate’ macrophages during EGFR-TKI resistance progression warrants further investigation in future studies.

While these mechanistic questions remain open, the consistent directionality of our experimental findings prompted us to examine the clinical relevance of the NSUN2/TRIM29/macrophage axis. We identified a potential correlation among NSUN2, TRIM29, and macrophage infiltration in patient specimens. Together with our mechanistic findings that NSUN2/TRIM29 promotes EGFR-TKI resistance through macrophage modulation, these clinical observations raise the possibility that targeting this axis may restore M1 macrophage infiltration and resensitize resistant tumors to EGFR-TKI, thereby offering a potential strategy to overcome acquired drug resistance. However, due to the limited sample size and the retrospective nature of the current analysis, the clinical correlation between NSUN2/TRIM29 and macrophage infiltration remains preliminary and requires cautious interpretation. Future prospective studies with larger patient cohorts, particularly those with well-documented EGFR-TKI treatment histories, are warranted to validate the clinical association among NSUN2, TRIM29, macrophage infiltration, and EGFR-TKI responsiveness, and to determine whether pharmacological inhibition of this axis could serve as an effective adjuvant approach to reverse EGFR-TKI resistance in NSCLC.

## Conclusions

In summary, the present study reveals that NSUN2-mediated EGFR-TKI resistance is critically immune-dependent, with NSUN2 expression dictating the immunological status of the NSCLC tumor microenvironment. Mechanistically, NSUN2 promotes immune evasion and EGFR-TKI resistance by suppressing macrophage infiltration and M1 polarization through RNA m^5^C-dependent upregulation of TRIM29, thereby establishing an immunosuppressive microenvironment. These findings identify NSUN2 as a pivotal epigenetic regulator and promising therapeutic target for overcoming EGFR-TKI resistance through immune modulation.

## Materials and methods

### Cell lines and reagents

The human lung adenocarcinoma cell lines HCC4006 cells were obtained from American Type Culture Collection (ATCC). PC-9 and THP-1 were kindly gifted by Prof. Jian Ding (Shanghai Institute of Materia Medica, Shanghai, China). All the cell lines were cultured at 37°C in a humidified 5% CO_2_ incubator in RPMI-1640 medium supplemented with 10% fetal bovine serum (FBS) (Gibco). Gefitinib and osimertinib were purchased from Selleck Chemicals and dissolved to 10 mmol/L with DMSO as stock solutions for in vitro studies.

### Generation of acquired resistant cells

HCC4006 cells and PC-9 cells were exposed to gradually increasing concentrations of gefitinib and osimertinib respectively for 6 months, up to a dose of 1 μM. The established resistant cell lines were named HCC4006/GR and PC-9/AR, respectively, and maintained in a medium containing 1 μM drugs for selective pressure.

### RNA interference

Cells were seeded in 6-well plates at 40% confluence. After 24 h, cells were transfected with the indicated siTRIM29 oligonucleotides using Lipofectamine RNAiMAX (Invitrogen) according to the manufacturer’s instructions. Then, the cells were cultured for 48 h and harvested for transwell migration assay. The target sequences of siRNA oligonucleotides were as follows: siTRIM29: 5′-GUGCAUUGAUGAGCAAUUA-3′.

### Plasmids and transfection

pcDNA3.1-NSUN2-WT and PLKO.1-shNSUN2 plasmids were generously provided by Prof. Yun-Gui Yang (Beijing Institute of Genomics, Beijing, China). The plasmids PLKO.1-shTRIM29 and GV492-TRIM29 were purchased from GENECHEM (Shanghai, China). For lentiviral production, a second-generation packaging system comprising psPAX2 and pMD2.G (both from Addgene) was employed. Briefly, HEK293T packaging cells at approximately 60% confluence were transfected with the indicated plasmids using Lipofectamine 3000 (Invitrogen) following the manufacturer's protocol. Viral supernatants were harvested 48 h post-transfection, filtered through 0.45 µm membranes (Millipore), and applied to target cells supplemented with 6 µg/mL polybrene (Solarbio, China). Stable polyclonal populations were subsequently established by puromycin selection (Solarbio, China) for two weeks and validated by Western blot analysis. The shRNA targeting sequence used was: shNSUN2: 5′-GCTGGCACAGGAGGGAATATA-3′; shTRIM29: 5′-CCAAUGAGAAGGCCAUCCU-3′.

### Cell viability assay

Cells were plated at 5 × 10^3^ cells per well in 96-well plates containing RPMI-1640 medium supplemented with 10% FBS. Following overnight attachment, cells were treated with serial dilutions of gefitinib or osimertinib for 72 h. Cell viability was then assessed by adding 10 µL CCK-8 reagent (Vazyme, China) to each well and incubating at 37°C for 1 h. Absorbance at 450 nm was measured using an EnVision® multimode plate reader (PerkinElmer), and IC_50_ values were determined by fitting concentration-response curves using a four-parameter logistic regression model.

### Isolation and activation of peripheral blood mononuclear cells (PBMCs)

Human peripheral blood was obtained from healthy donors at the Henan Precision Clinical Pharmacy Laboratory, First Affiliated Hospital of Zhengzhou University. Briefly, peripheral blood was diluted with an equal volume of PBS and carefully layered onto Lymphoprep density gradient medium (STEMCELL, catalog no 07801) according to the manufacturer’s instructions. Following centrifugation, the PBMC layer at the interface was carefully aspirated, washed with PBS, and subjected to red blood cell lysis if necessary. The purified PBMCs were then resuspended in complete RPMI medium for culture. For T cell activation, plates were pre-coated with anti-CD3 antibody (Absin, abs160014, China) and incubated at 37°C for 2 h. Subsequently, anti-CD28 antibody (Absin, abs160015, China) and IL-2 (UA Bioscience, UA040057, China) were added, and cells were cultured for 48 h to achieve T cell activation.

### Tumor cell-T cell co-culture assay

For proliferation assessment, NSCLC cells were seeded into 96-well plates and allowed to adhere overnight. Activated PBMCs were subsequently added at a PBMC-to-tumor cell ratio of 4:1 and co-cultured for designated time periods. Following incubation, non-adherent cells (primarily PBMCs and dead cells) were removed, and adherent tumor cells were thoroughly washed. Cell viability was then determined using the CCK-8 assay according to the manufacturer's protocol, with results normalized to control wells without PBMC addition. All experiments were performed in triplicate.

For apoptosis analysis, NSCLC cells were seeded into 12-well plates and cultured overnight prior to the addition of activated PBMCs at a ratio of 4:1. After 48 h of co-culture, both suspended and adherent cell populations were harvested and subjected to Annexin V-FITC staining following the manufacturer's instructions. Stained samples were analyzed by flow cytometry (BD Accuri™ C6 Plus), gating on intact tumor cells based on forward and side scatter properties (FSC/SSC). The percentage of viable and apoptotic tumor cells was quantified, with tumor cells cultured in the absence of PBMCs serving as controls.

### Quantitative real-time PCR (qRT-PCR)

The total RNA was extracted using TRIzol reagent (Invitrogen) and reversely transcribed to complementary DNA (cDNA) using iScript cDNA Synthesis Kit (Bio-Rad). Real-time PCR was performed using iTaq Univer SYBR Green Supermix (Bio-Rad) according to the manufacturer’s instructions. GAPDH or 18S rRNA was used as a housekeeping gene for normalization. Results were represented as fold expression. The primer pairs used for qRT-PCR analysis were listed in the Supplementary Table 1.

### Macrophage migration assay

Macrophage migration was assessed using transwell assay. Briefly, PMA-activated THP-1 cells (1.0 × 10^6^ cells/mL) cells in serum-free medium were added to the upper chamber (3422, Corning), and conditioned medium from cancer cell was added to the lower chamber as the chemoattractant. The migrated cells on the lower surface were fixed with 4% paraformaldehyde, stained with 0.5% crystal violet, and counted under an optical microscope after 24 h.

### Flow cytometry analysis of macrophage markers

Conditioned medium was generated by culturing cells in serum-free medium for 48 h, followed by collection, centrifugation, filtration, and supplementation with 10% FBS. For surface marker detection, cells were treated with conditioned medium, washed with PBS, detached using EDTA- and phenol red-free trypsin, and harvested in complete medium. After centrifugation at 800 rpm for 8 min at 4°C, cells were resuspended in flow cytometry staining buffer and incubated with fluorochrome-conjugated CD86 antibody or isotype control at 4°C in the dark for 30 min. For intracellular marker detection, cells were additionally fixed and permeabilized using Fix and Permeabilize solution at 4°C in the dark for 30 min, washed with 1 × Perm/Wash buffer, and incubated with fluorochrome-conjugated CD206 antibody or isotype control at 4°C in the dark for 30 min. All stained cells were washed, resuspended in staining buffer, and analyzed by flow cytometry within 1 h. Data were analyzed using FlowJo software, with macrophage subpopulations identified by gating on CD86⁺ or CD206⁺ cells within the live cell population.

### Western blotting analysis

Total cellular protein was isolated using RIPA lysis buffer (Beyotime, China) containing protease inhibitors (Roche) and phosphatase inhibitor cocktail (Sigma), and protein concentrations were quantified with the BCA Protein Assay Kit (Beyotime, China). Protein lysates were separated by SDS-PAGE and electrotransferred onto nitrocellulose membranes. After blocking, membranes were incubated with primary antibodies followed by horseradish peroxidase-conjugated secondary antibodies. Immunoreactive bands were detected using enhanced chemiluminescence reagents (Thermo Scientific). The following primary antibodies were used: anti-NSUN2 (Sigma, HPA037896) and anti-GAPDH (Cloud Clone, CAB932Mi01).

### RNA sequencing and bioinformatics analysis

RNA-seq libraries were constructed from 20 to 30 ng mRNA using the KAPA Stranded mRNA-Seq Kit (Illumina platform) according to the manufacturer's protocol, followed by paired-end sequencing on the Illumina HiSeq-PE150 instrument. Raw reads were processed with FastQC for quality control, adapter trimming with cutadapt, and removal of low-quality bases using Trimmomatic. Clean reads were aligned to the hg38 reference genome with Ensembl 78 annotation using HISAT2 with default parameters. Gene-level read counts were generated using HTSeq-count with parameters -m union -s no Gene expression was quantified as TPM using DESeq2, and differentially expressed genes were identified with thresholds of |FoldChange| ≥ 1.2 and adjusted p-value < 0.05.

### RNA-BisSeq library preparation and bioinformatics analysis

Following a published protocol with minor modifications [31], we performed RNA fragmentation and bisulfite conversion. Purified mRNA was supplemented with Luciferase control RNA at a 300:1 ratio to assess bisulfite conversion efficiency. Library construction was carried out using the KAPA Stranded mRNA-Seq Kit (Illumina) according to the manufacturer’s protocol, followed by paired-end 150 bp sequencing on the Illumina HiSeq platform. For bioinformatics processing, raw reads were subjected to quality control using FastQC, applying the same procedures described in the ‘RNA-Seq bioinformatics analyses’ section. Processed reads longer than 35 nucleotides were mapped to the human reference genome (hg38) with meRanGs, a splice-aware RNA-BSseq aligner available in the meRanTK package. Samples demonstrating C-to-T conversion rates above 99% were removed from further analysis. Genome-wide m^5^C calling was executed using meRanCall with the following parameters:mBQ 20 -mr 0.1. Stringent filtering criteria were applied to retain high-confidence m5C sites: total coverage depth ≥20 (combining methylated and unmethylated cytosine counts), methylated cytosine depth ≥3, and methylation level ≥0.1 in at least 50% of replicates within each experimental condition. BEDTools intersectBed was employed for genomic annotation of m5C positions. Sites exhibiting an absolute methylation difference >0.05 between comparison groups were classified as differentially methylated. For functional interpretation, clusterProfiler was utilized to perform GO and KEGG pathway enrichment analysis on genes with significant differential methylation, with statistical significance defined as adjusted p-value <0.05.

### Animal studies

The male NCG nude mice (5-6 weeks old) were purchased from GemPharmatech Co., Ltd. (Jiangsu, China) and maintained under specific pathogen-free conditions. For xenograft implantation, PC-9/AR-shCtrl, PC-9/AR-shNSUN2 (6.0 × 10^6^ cells/100 µL) were suspended in PBS and subcutaneously injected into the right flank of all mice. Tumor volume was measured every 3 days and calculated by caliper measurements of the width (W) and length (L) of each tumor using the following formula: V= (L × W^2^)/2. Mice were sacrificed and tumors were collected for further analysis.

For immune system reconstitution model: PBMC from healthy donors were activated and expanded as described above. Three day before tumor cell injection, PBMC (1 × 10^7^ cells) was adoptively transferred to NCG mice via the tail vein. When tumors formed and reached a volume of ≥100 mm^3^, mice were administrated with 5 mg/kg osimertinib (S7297, selleck, China) or 0.5% CMC—Na via gavage once daily for 2 weeks. At the end, the fresh spleen was isolated and subjected to flow cytometry for detecting T-cell percentage. All animal experiments were approved by the Institutional Animal Care and Use Committee of the First Affiliated Hospital of Zhengzhou University (approval number: 2024-KY-0712).

### Clinical tissue samples

A single NSCLC tissue microarray was constructed for this study, incorporating 32 formalin-fixed paraffin-embedded (FFPE) tissue cores comprising both tumor and adjacent non-tumor regions. These specimens were prospectively collected from consecutive NSCLC patients who had undergone curative resection at the First Affiliated Hospital of Zhengzhou University (Zhengzhou, China). The study protocol was approved by the Ethics Committee of the First Affiliated Hospital of Zhengzhou University (approval no 2024-KY-0712), and written informed consent was obtained from every participant.

### Immunohistochemistry staining analysis

Immunohistochemistry (IHC) staining was performed at Servicebio Technology (Wuhan, China). Paraffin-embedded tissue sections were baked at 60°C for 4 h, followed by deparaffinization using BioDewax and Clear Solution (ServiceBio, China). Antigen retrieval was performed by heating sections in citric acid buffer (pH 6.0, ServiceBio, China) at 95°C for 23 min. Endogenous peroxidase activity was quenched with 3% hydrogen peroxide in the dark at room temperature for 25 min. Sections were then blocked with 3% BSA for 30 min at room temperature and incubated with primary antibody overnight at 4°C according to the manufacturer's recommendations. After washing, sections were incubated with horseradish peroxidase-conjugated secondary antibodies at room temperature for 50 min. Stained slides were scanned using the 3DHISTECH PANNORAMIC VIEWER. For staining quantification, the Aipathwell software (ServiceBio, China) was used for digital photograph analysis of antigen expression. Primary antibodies were selected according to the experimental application. Anti-NSUN2 (Sigma, HPA037896) was employed for animal xenograft tumor tissues. For human tissue microarray analysis, anti-NSUN2 (Proteintech, 20854-1-AP), anti-TRIM29 (Proteintech, 17542-1-AP), and anti-CD68 (Servicebio Technology, GB153150) were utilized.

### Statistical analysis

Data are expressed as mean ± standard deviation (SD) unless otherwise specified. Comparisons between groups were performed using two-tailed Student’s t-test. Statistical significance was defined as *p < 0.05.

## Funding

This study was supported by grants from the National Natural Science Foundation of China (No. 32570703, 81702253), the Science and Technology Innovation Leading Talent Program of Henan Province (No. 254200510008), the Scientific Research and Innovation Team of The First Affiliated Hospital of Zhengzhou University (No. ZYCXTD2023010), the Key Scientific Research Project of the Education Department of Henan Province (No. 26A350013), and the Medical Science and Technique Foundation of Henan Province (No. 262102310250, 252102310192).

## Ethics approval and consent to participate

This study was approved by the Institutional Review Board of the First Affiliated Hospital of Zhengzhou University (the approval number 2024-KY-0712) and complied with the Declaration of Helsinki. All patients and healthy volunteers provided the written informed consent according to the institutional guidelines. Ethical approvals for the animal experiments were obtained from the Ethics Committee of the First Affiliated Hospital of Zhengzhou University (Zhengzhou, China).

## Data availability

The raw sequencing data generated in this study, including RNA-seq and RNA-BisSeq, have been deposited in the Genome Sequence Archive (GSA) with the accession number HRA018854 (linked to BioProject PRJCA065747). All other relevant data that support the findings are available from the corresponding author upon reasonable request.

## CRediT authorship contribution statement

**Yueqin Wang:** Writing – review & editing, Writing – original draft, Visualization, Supervision, Funding acquisition, Conceptualization. **Jingyao Wei:** Writing – review & editing, Software, Funding acquisition, Data curation. **Wenbin Xu:** Writing – review & editing, Methodology, Investigation, Data curation. **Yu Zhang:** Writing – review & editing, Validation, Methodology, Investigation. **Luyao Feng:** Writing – review & editing, Validation, Methodology, Investigation, Data curation. **Lizhen Zhang:** Writing – review & editing, Validation, Methodology, Investigation, Data curation. **Shuaibing Liu:** Writing – review & editing, Methodology, Investigation, Data curation. **Xin Tian:** Writing – review & editing, Supervision, Funding acquisition.

## Declaration of competing interest

The authors declare no competing interests.

## Appendix Supplementary materials

Image, application 1

Image, application 2

## References

[1] Bray F., Laversanne M., Sung H., Ferlay J., Siegel R.L., Soerjomataram I., Jemal A. (2024). Global cancer statistics 2022: GLOBOCAN estimates of incidence and mortality worldwide for 36 cancers in 185 countries. CA Cancer J. Clin..

[2] Berns A. (2024). Transforming lung cancer types. Science.

[3] Yang J.C., Lu S., Hayashi H., Felip E., Spira A.I., Girard N., Kim Y.J., Lee S.H., Ostapenko Y., Danchaivijitr P., Liu B., Alip A., Korbenfeld E., Mourão Dias J., Besse B., Passaro A., Lee K.H., Xiong H., How S.H., Cheng Y., Chang G.C., Yoshioka H., Thomas M., Nguyen D., Ou S.I., Mukhedkar S., Prabhash K., D'Arcangelo M., Alatorre-Alexander J., Vázquez Limón J.C., Alves S., Stroyakovskiy D., Peregudova M., Şendur M.A.N., Yazici O., Califano R., Gutiérrez Calderón V., de Marinis F., Kim S.W., Gadgeel S.M., Owen S., Xie J., Sun T., Mehta J., Venkatasubramanian R., Ennis M., Fennema E., Daksh M., Roshak A., Man J., Knoblauch R.E., Bauml J.M., Baig M., Shah S., Sethi S., BC Cho (2025). MARIPOSA investigators. Overall survival with Amivantamab-Lazertinib in EGFR-mutated advanced NSCLC. N. Engl. J. Med..

[4] Jänne P.A., Planchard D., Kobayashi K., Yang J.C., Liu Y., Valdiviezo N., Kim T.M., Jiang L., Kagamu H., Yanagitani N., Wang J., Biswas B., Poltoratskiy A., Neron Y., Rojas C., Koubkova L., Escriu C., Ezeife D.A., Mann H., Armenteros-Monterroso E., Rukazenkov Y., Lee C.K. (2026). FLAURA2 Investigators. Survival with Osimertinib plus chemotherapy in EGFR-mutated advanced NSCLC. N. Engl. J. Med..

[5] Lee S.H., Lu S., Hayashi H., Felip E., Spira A.I., Girard N., Kim Y.J., Ostapenko Y., Danchaivijitr P., Liu B., Alip A., Korbenfeld E., Dias J.M., Lee K.H., Xiong H., How S.H., Cheng Y., Chang G.C., Chih-Hsin Yang J., Besse B., Thomas M., Shah S., Baig M., Curtin J.C., Zhang J., Xie J., Sun T., Sethi S., Wang M., Fennema E., Daksh M., Ennis M., Bauml J.M., Cho B.C (2025). Lazertinib versus Osimertinib in previously untreated EGFR-mutant advanced NSCLC: a randomized, double-blind, exploratory analysis from MARIPOSA. J. Thorac. Oncol..

[6] Zhao J., Xu W., Zhou F., Zhang X., Zhou M., Miao D., Yu L., Zhang Y., Fan J., Zhou C., Li W., Mok T., Le X., Li M., Xia Y. (2026). Navigating the landscape of EGFR TKI resistance in EGFR-mutant NSCLC - mechanisms and evolving treatment approaches. Nat. Rev. Clin. Oncol..

[7] Kirchner M., Kluck K., Brandt R., Volckmar A.L., Penzel R., Kazdal D., Endris V., Neumann O., Seker-Cin H., Goldschmid H., Glade J., Allgäuer M., Kriegsmann M., Winter H., Muley T., Perner S., Frost N., Reck M., Fröhling S., Schirmacher P., Thomas M., Budczies J., Christopoulos P., Stenzinger A. (2021). The immune microenvironment in EGFR- and ERBB2-mutated lung adenocarcinoma. ESMO Open..

[8] Yang L., He Y.T., Dong S., Wei X.W., Chen Z.H., Zhang B., Chen W.D., Yang X.R., Wang F., Shang X.M., Zhong W.Z., Wu Y.L., Zhou Q. (2022). Single-cell transcriptome analysis revealed a suppressive tumor immune microenvironment in EGFR mutant lung adenocarcinom. J. Immunother. Cancer.

[9] Isomoto K., Haratani K., Hayashi H., Shimizu S., Tomida S., Niwa T., Yokoyama T., Fukuda Y., Chiba Y., Kato R., Tanizaki J., Tanaka K., Takeda M., Ogura T., Ishida T., Ito A., Nakagawa K. (2020). Impact of EGFR-TKI treatment on the tumor immune microenvironment in EGFR mutation-positive non-small cell lung cancer. Clin. Cancer Res..

[10] Peng S., Wang R., Zhang X., Ma Y., Zhong L., Li K., Nishiyama A., Arai S., Yano S., Wang W. (2019). EGFR-TKI resistance promotes immune escape in lung cancer via increased PD-L1 expression. Mol. Cancer.

[11] Hu S., Shi X., Hu Q., Wang X., Zhou G. (2026). Immunotherapy in EGFR-TKI-resistant NSCLC: mechanisms, therapeutic strategies, and emerging challenges. Biochim. Biophys. Acta Rev. Cancer.

[12] Zhang Y., Dai X., Liang J., Yang C., Wan C., Sun L., Qiao Q., Li S., Bao X., Chen X., Huang L., Liu X., Zhang S., Zhou C., Gu X., Zhou F., Yang K., Wen L., Lu Y., Zhang H. (2025). Immunotherapy in EGFR-mutant NSCLC after TKI resistance: role of mutation subtypes and progression patterns. Lung Cancer.

[13] Cao X., Geng Q., Fan D., Wang Q., Wang X., Zhang M., Zhao L., Jiao Y., Deng T., Liu H., Zhou J., Jia L., Xiao C. (2023). m6A methylation: a process reshaping the tumour immune microenvironment and regulating immune evasion. Mol. Cancer.

[14] Li P., Huang D. (2024). NSUN2-mediated RNA methylation: molecular mechanisms and clinical relevance in cancer. Cell Signal..

[15] Jiang T., Jiang N., Chen X., Xiong Z. (2025). The role of NSUN Family genes in m5C methylation and diseases. Biomedicine.

[16] Yang Y., Cao L., Xu X., Li D., Deng Y., Li L., Zeng B., Jiang H., Shan L., Huang Y., Xu Y., Ma L. (2025). NSUN2/ALYREF axis-driven m5C methylation enhances PD-L1 expression and facilitates immune evasion in non-small-cell lung cancer. Cancer Immunol. Immunther..

[17] Li Z., Xue H., Li J., Zheng Z., Liu Z., Dong X., Wang H., Chen J., Xu S. (2024). CDKL1 potentiates the antitumor efficacy of radioimmunotherapy by binding to transcription factor YBX1 and blocking PD-L1 expression in lung cancer. J. Exp. Clin. Cancer Res..

[18] Wang Y., Wei J., Feng L., Li O., Huang L., Zhou S., Xu Y., An K., Zhang Y., Chen R., He L., Wang Q., Wang H., Du Y., Liu R., Huang C., Zhang X., Yang Y.G., Kan Q., Tian X. (2023). Aberrant m5C hypermethylation mediates intrinsic resistance to gefitinib through NSUN2/YBX1/QSOX1 axis in EGFR-mutant non-small-cell lung cancer. Mol. Cancer.

[19] Lu C., Gao Z., Wu D. (2024). J. Immunother. Cancer.

[20] Patel S.A., Nilsson M.B., Yang Y., Le X., Tran H.T., Elamin Y.Y., Yu X., Zhang F., Poteete A., Ren X., Shen L., Wang J., Moghaddam S.J., Cascone T., Curran M., Gibbons D.L., Heymach J.V. (2023). IL6 Mediates suppression of T- and NK-cell function in EMT-associated TKI-resistant EGFR-mutant NSCLC. Clin. Cancer Res..

[21] Liu Y., Yu Y., Hu C., Jiang M., Zhao C., Li X., Cheng L., Zhou C. (2025). ZEB2 upregulation modulates the polarization of TAMs toward the immunosuppressive state in EGFR-TKI-resistant NSCLC. Cancer Drug Resist..

[22] Jeong H.O., Lee H., Kim H., Jang J., Kim S., Hwang T., Choi D.W., Kim H.S., Lee N., Lee Y.M., Park S., Jung H.A., Sun J.M., Ahn J.S., Ahn M.J., Park K., Lee S., Lee S.H (2022). Cellular plasticity and immune microenvironment of malignant pleural effusion are associated with EGFR-TKI resistance in non-small-cell lung carcinoma. iScience.

[23] Mouri A., Kaira K., Yamaguchi O., Shiono A., Miura Y., Hashimoto K., Yamasaki S., Nishihara F., Imai H., Kobayashi K., Kagamu H. (2025). Correlation between T cell immunity and the duration of EGFR-TKI resistance acquisition in patients harboring EGFR mutations. Cancer Immunol. Immunther..

[24] Yuan S., Chen W., Yang J., Zheng Y., Ye W., Xie H., Dong L., Xie J. (2022). Tumor-associated macrophage-derived exosomes promote EGFR-TKI resistance in non-small cell lung cancer by regulating the AKT, ERK1/2 and STAT3 signaling pathways. Oncol. Lett..

[25] Fang H., Wang H., Liu X., Deng Y., Zhao L., Xu K., Ye L., Liu Y., Zhao W., Zhou Y., Li Q., He Y. (2026). HMOX1 expression influence the role of macrophage in EGFR-TKI resistance of lung adenocarcinoma. Int. Immunopharmacol..

[26] Meng Q., Xie Y., Sun K., He L., Wu H., Zhang Q., Liang T. (2024). ALYREF-JunD-SLC7A5 axis promotes pancreatic ductal adenocarcinoma progression through epitranscriptome-metabolism reprogramming and immune evasion. Cell Death. Discov..

[27] Lv Z., Xue C., Zhang L., Sun J., Bo C. (2021). Elevated mRNA level of Y-box binding protein 1 indicates unfavorable prognosis correlated with macrophage infiltration and T cell exhaustion in luminal breast cancer. Cancer Manag. Res..

[28] Satoh T., Takeuchi O., Vandenbon A., Yasuda K., Tanaka Y., Kumagai Y., Miyake T., Matsushita K., Okazaki T., Saitoh T., Honma K., Matsuyama T., Yui K., Tsujimura T., Standley D.M., Nakanishi K., Nakai K., Akira S. (2010). The Jmjd3-Irf4 axis regulates M2 macrophage polarization and host responses against helminth infection. Nat. Immunol..

[29] Yang X., Wang X., Liu D., Yu L., Xue B., Shi H. (2014). Epigenetic regulation of macrophage polarization by DNA methyltransferase 3b. Mol. Endocrinol..

[30] Zhu Y., Knolhoff B.L., Meyer M.A., Nywening T.M., West B.L., Luo J., Wang-Gillam A., Goedegebuure S.P., Linehan D.C., DeNardo D.G. (2014). CSF1/CSF1R blockade reprograms tumor-infiltrating macrophages and improves response to T-cell checkpoint immunotherapy in pancreatic cancer models. Cancer Res..

[31] Yang X., Yang Y., Sun B.F., Chen Y.S., Xu J.W., Lai W.Y., Li A., Wang X., Bhattarai D.P., Xiao W., Sun H.Y., Zhu Q., Ma H.L., Adhikari S., Sun M., Hao Y.J., Zhang B., Huang C.M., Huang N., Jiang G.B., Zhao Y.L., Wang H.L., Sun Y.P., Yang Y.G (2017). 5-methylcytosine promotes mRNA export-NSUN2 as the methyltransferase and ALYREF as an m^5^C reader. Cell Res..

