# Supplementary material for: Tumor cell-intrinsic NSUN2 deficiency reprograms macrophages to sensitize non-small cell lung cancer to EGFR inhibitors by reversing immune evasion
Source: Neoplasia. 2026 Jun 12;79:101326. doi: 10.1016/j.neo.2026.101326 (PMC13276385; doi:10.1016/j.neo.2026.101326)
Supplement: Supplementary file 1 [file mmc1.docx]

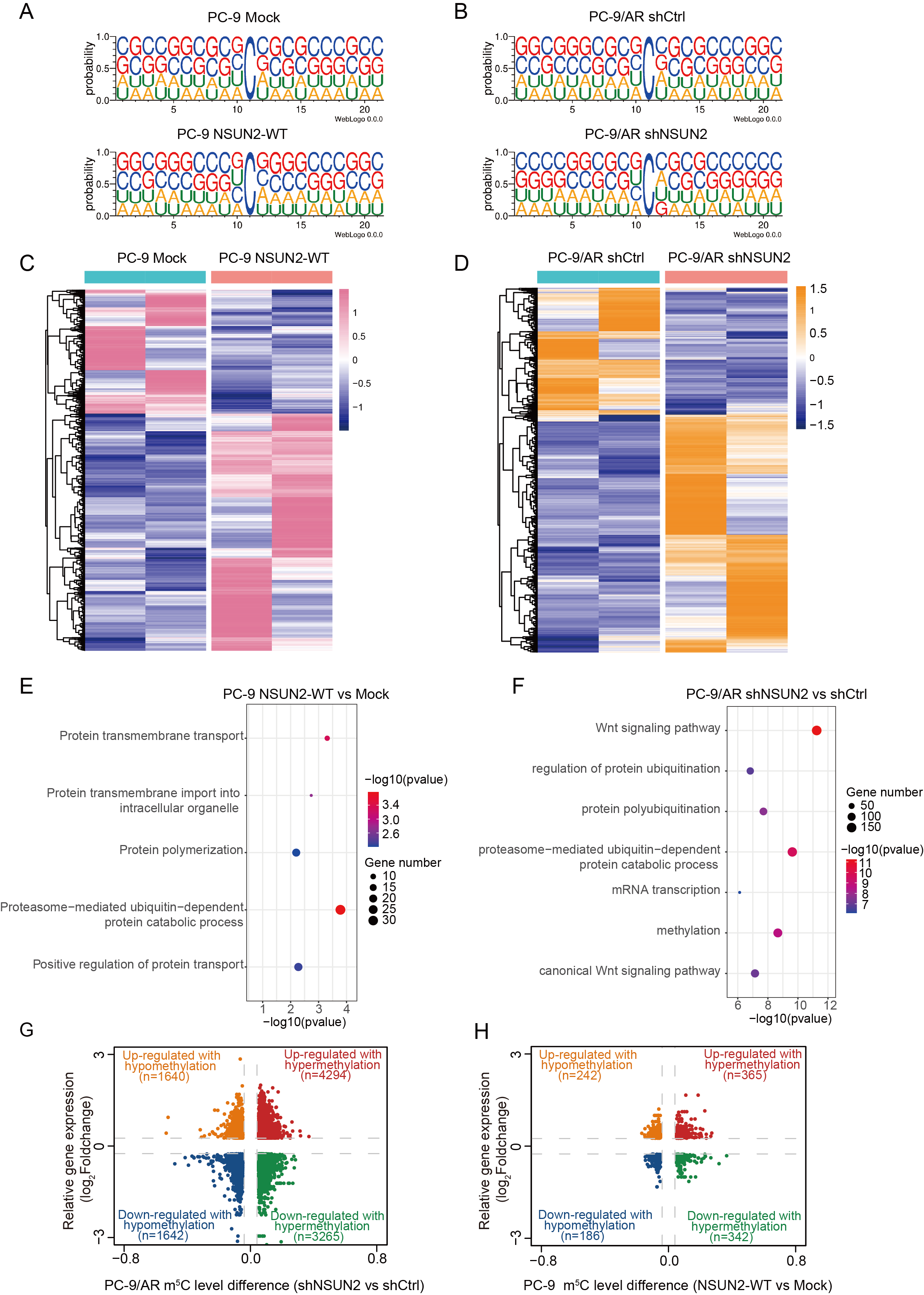


**Supplemental Figure 1 NSUN2 modulates m⁵C methylation patterns and downstream signaling pathways in NSCLC cells**. (A, B) Sequence motif identified from sequencing profile in PC-9 cells (A) and PC-9/AR cells (B). (C, D) Heatmap showing the differential m^5^C methylation change in PC-9 cells with NSUN2 overexpression (NSUN2-WT) (C) and PC-9/AR cells with NSUN2 knockdown (shNSUN2) (D). (E, F) KEGG analysis of genes with m^5^C hypomethylation in PC-9 cells with NSUN2 overexpression (NSUN2-WT) (E) and PC-9/AR cells with NSUN2 knockdown (shNSUN2) (F). (G, H) Significant changes in the m^5^C methylation and mRNA expression levels in PC-9/AR shNSUN2 cells (G) and PC-9 NSUN2-WT cells (H).
